# Spontaneous regression in alveolar soft part sarcoma: case report and literature review

**DOI:** 10.1186/1477-7819-7-53

**Published:** 2009-06-10

**Authors:** Mohammed N BaniHani, Abdel Rahman A Al Manasra

**Affiliations:** 1 King Abdullah University Hospital, Department of Surgery, Jordan University of Science and Technology, Irbid, Jordan

## Abstract

**Background:**

Sarcomas are a type of malignant tumors that arise from connective tissue. They are most of the time found in extremities

**Case presentation:**

We are presenting a case of adult male patient, who was found to have huge abdominal mass and multiple gastric and duodenal polyps. Pathological diagnosis for all lesions was Alveolar soft part sarcoma. Although he complained from metastasis to both lungs and right atrium, all these deposits regressed spontaneously. Patient stated that he used some herbs (Teucrium polium, Cat Thyme) prescribed for him. No chemotherapy or radiotherapy was given. The duration of regression was about 5 months before other lesions appeared. Later on, he died secondary to brain metastasis.

**Conclusion:**

ASPS is a rare type of sarcomas that affect primarily the lower limbs. This tumor does rarely metastasize to the gastrointestinal tract.

## Background

Sarcomas are a type of malignant tumors that arise from connective tissue. They are most of the time found in extremities. The term -Soft Part- is used to distinguish these sarcomas from bone sarcomas. Although soft tissue sarcomas are linked to the tissue of origin; alveolar description is based on the histological pattern of the tumor rather than the origin.

## Case presentation

A 38 year old male married patient, medically free, referred to our center from a another hospital complaining from lower abdominal pain of few months duration, intermittent, vague, gripping in nature, associated with abdominal distention.

He didn't undergo any type of surgery previously. Past history of the patient was negative for any fever, sweating, vomiting, constipation, or weight changes. He also didn't suffer from obstructive urinary symptoms. Family history was negative too.

On examination, he actually was found to have large left, hypochondrial mass. It was painless & hard in consistency.

**Figure 1:**
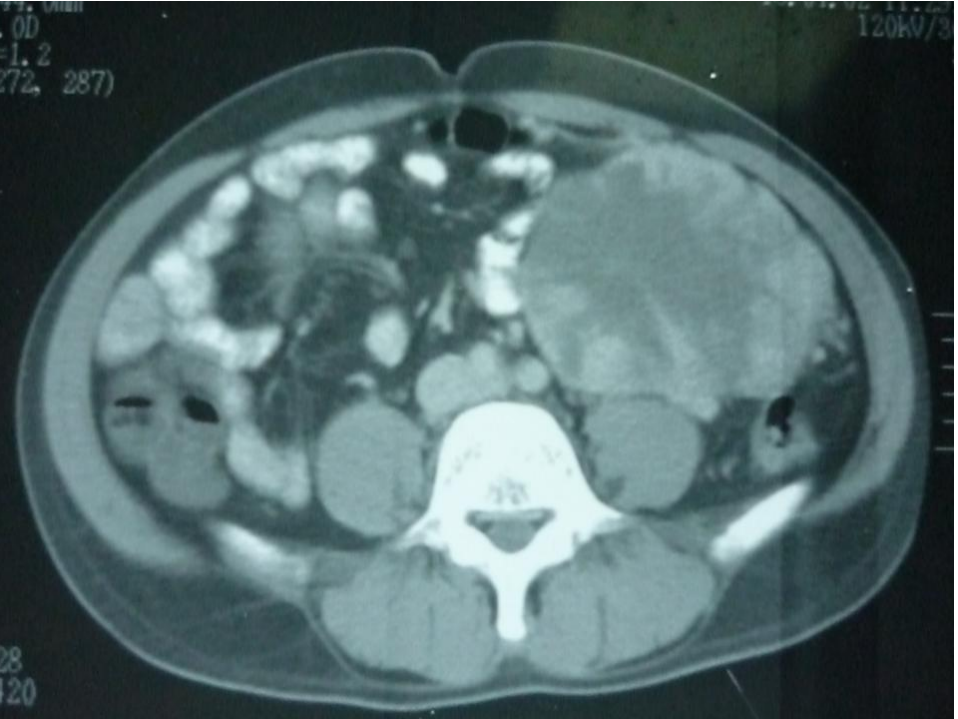
**Large abdominal mass**.

**Figure 2:**
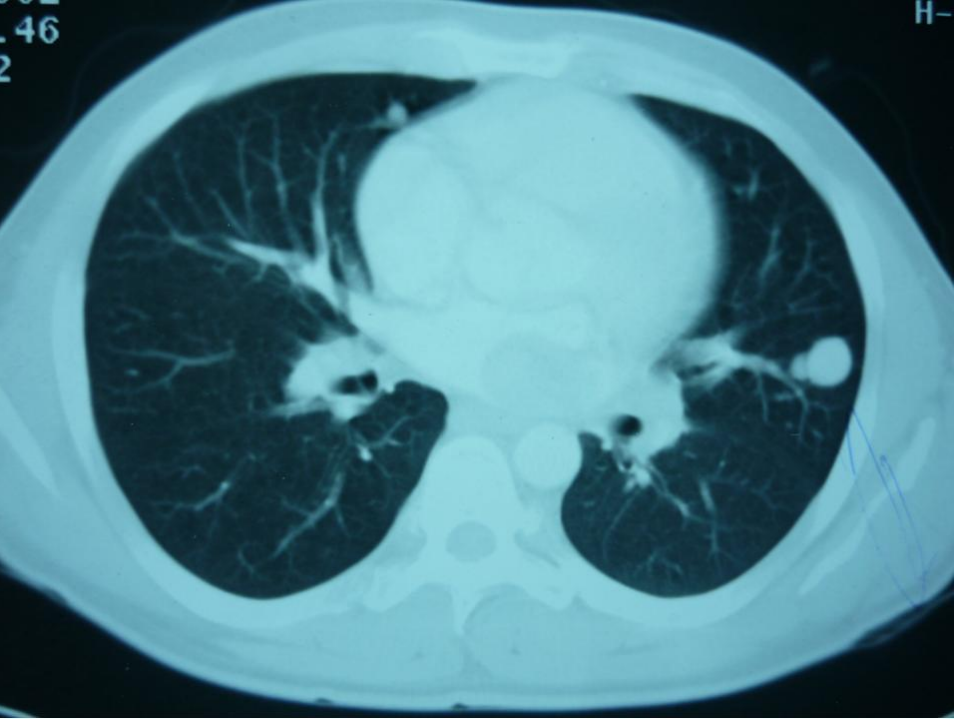
**Lung metastasis**.

**Figure 3:**
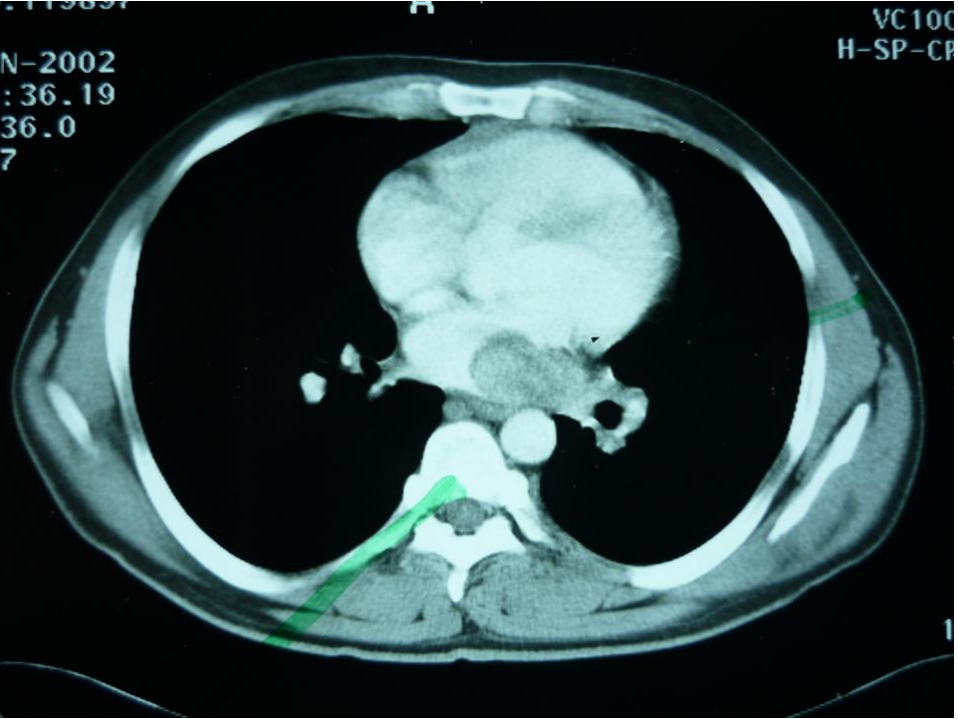
**Atrial lesion**.

**Figure 4:**
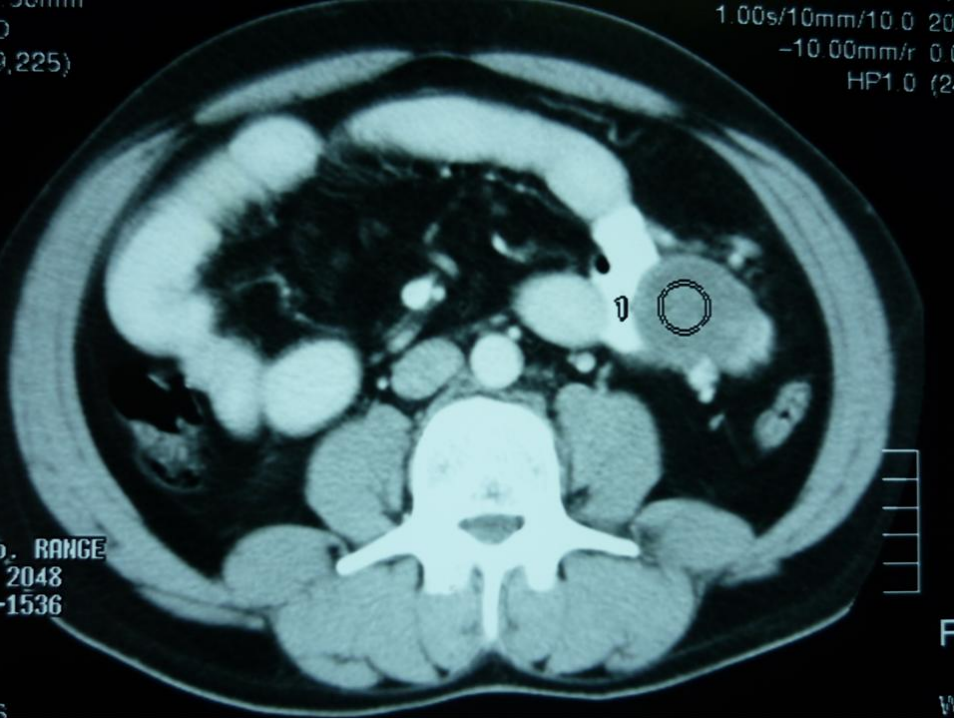
**Abdominal tumor regression**.

**Figure 5:**
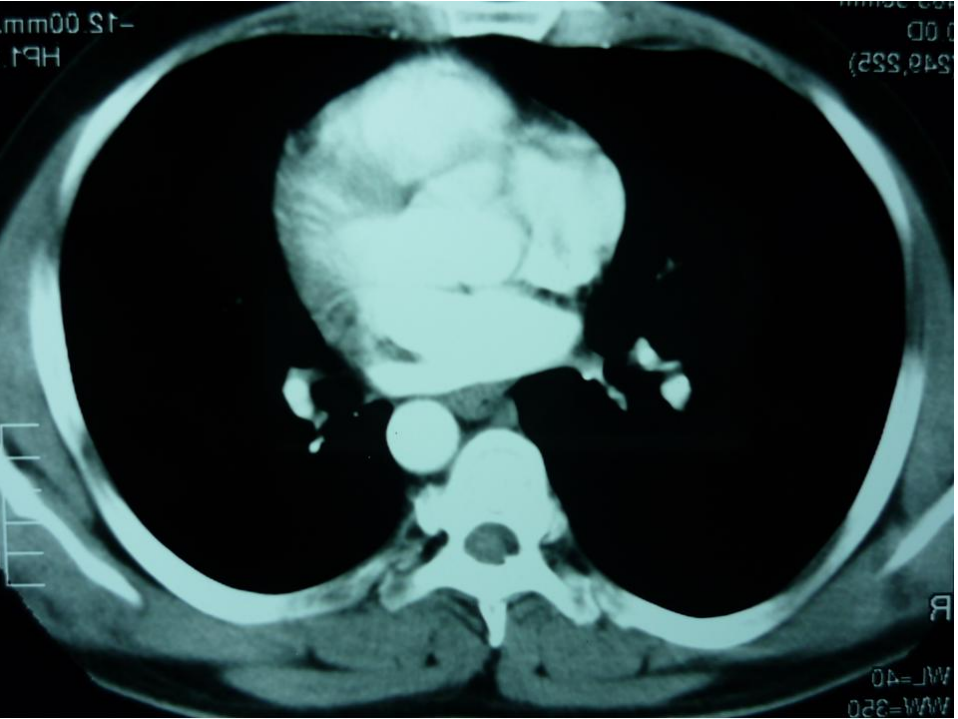
**Lung and cardiac metastasis regression**.

**Figure 6:**
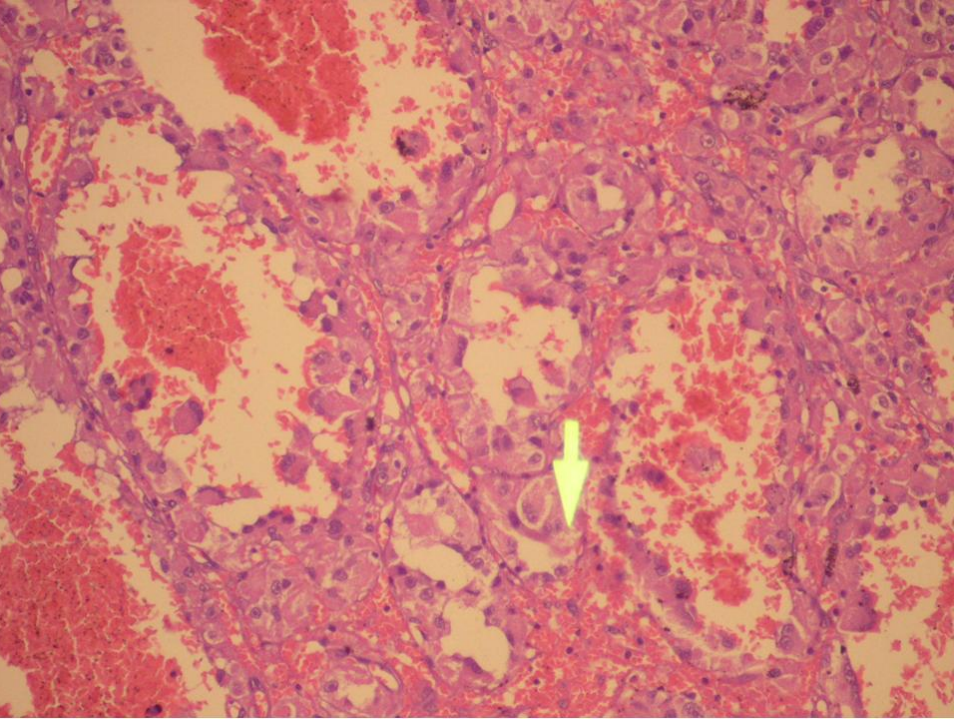
**Histologic view**.

On investigation, his hemoglobin level and leukocytic count were within normal range. Markers for colon, liver and prostate cancers were negative. On computerized tomographic (CT) scan; there was a large heterogeneous mass measuring about 16 × 12 × 10 cm occupying the left hypochondrial region with multiple small metastatic right lung lesions (figures 1 &2). CT guided fine needle aspiration was performed and was positive for malignancy. At that time, patient was offered surgical intervention for the abdominal mass but he refused any further treatment. Upon follow up of the patient, CT scan was repeated 7 months later, there were multiple bilateral lung deposits the largest measuring about 1 cm in diameter. Also there was a large tumor in left atrium (figure 3) extending to inferior left pulmonary vein measuring about 2 cm. the mass in the abdomen was still there as before. Then patient was sent home for tender care upon his request. Five months spent at home and the man came back again. He just reported a better feeling than before upon using some herbs (Teucrium polium, Cat Thyme) prescribed for him. On examination; there was no masses felt in the abdomen, so that CT scan was repeated. Unexpectedly; there was more than 60% reduction in the tumor size as well as disappearance of lungs & heart lesions as illustrated by figures 4 &5. During this interval, he was not given any type of radiation or chemicals as a therapy for his illness. Surgery was offered again and the mass removed by laparoscopic approach. Histopathologic analysis described a rounded hemorrhagic, necrotic and partially hyalized tumor that is markedly vascular and has a very prominent alveolar pattern. Immunohistochemical staining including cytokeratin, desmin, actin, S-100, synaptophysin, HMB45, chromogranin, vimentin, myoglobin and PAS was performed. All stains were negative apart from PAS and myoglobin which were positive (figure 6). The overall picture was consistent with alveolar soft part sarcoma (ASPS) infiltrating the omentum. Similarly, endoscopic assessment of upper gastrointestinal tract (due to epigastric pain) demonstrated multiple sessile polyps in stomach & duodenum; biopsies were taken, and the diagnosis was alveolar soft part sarcoma. Few months' later, patient developed headaches, brain CT scan was done and unfortunately he was found to have metastatic brain lesions. He was referred to a specialized center for radiotherapy and died within 6 months.

## Discussion

ASPS is a rare type of sarcomas, accounting for less than 1% of soft tissue sarcomas [1]. It was firstly described by Christopherson et al in 1952 [2]. The usual age for presentation is 15–35 years. The most common site for the tumor is lower extremities [3], representing about 60% of ASPS cases. Other sites included are head and neck, more commonly in children.

It has been reported that certain organs can be very unusually targeted by ASPS malignant cells. Part of the list includes female genital tracts, breasts and mediastinum. [4-7]

Gastrointestinal tract is also rarely involved by these malignancies. Only 3 cases were reported. In 2001, Michael S.et al reported a case of ASPS metastatic to small bowel mucosa causing polyposis and intussuseption in a 42 year old male with long history of ASPS metastasis to lungs and brain. [3] Later on; in 2003, Zilber S. et al reported another 43 year old woman who had a leg primary tumor more than 15 years ago and multiple lung and brain metastases. She also was found to have caecal metastases, revealed by an anemia, she was treated by laparoscopic right colectomy [8]. Primary Gastrointestinal ASPS is extremely rare. Only one case was reported in 2000 by Yaziji H et al. He described a Primary ASPS of the stomach in a 54 year old Italian woman without evidence of primary neoplasm elsewhere ten years following the initial diagnosis [9].

In our case, screening for primary lesions in extremities was negative. Pathological wise, depending on the cellularity of the tumor, the primary source is more likely to be from mesenchymal cells of the mesentery than from gastric or duodenal polyps,. This means that it can be considered the first case of this origin type to be reported in the literature.

Spontaneous regression is a term that is used interchangeably with spontaneous remission. Tilden Everson and Warren Cole have defined spontaneous regression of cancer as the partial or complete disappearance of a malignant tumor in the absence of all treatment or in the presence of therapy which is considered inadequate to exert a significant influence on neoplastic disease. They stated that it is not implied that spontaneous regression need progress to complete disappearance of tumor nor that spontaneous regression is synonymous with cure, all cases in which a tumor underwent apparent spontaneous regression in one area but flourished unchecked in other areas or reappeared at a later date are considered as valid examples of this phenomenon in their opinion. Until now, this definition of spontaneous regression, with few additions, has remained the one in most common usage today [10]. In fact, what happened in our patient case does fit to the criteria mentioned in their definition of spontaneous regression.

The highest incidence of this phenomenon was in tumors of genitourinary organs. According to Caryle Hirshberg [11], between 1918 and 1993, a total of 64 soft tissue sarcomas cases were reported to undergo spontaneous regression, however, spontaneous regression in the more specific alveolar pattern sarcomas has not been identified during that period.

To the best of our knowledge, only a single case of ASPS with spontaneous regression (of pulmonary metastases) has been published. In 2003, A 14 year old male patient was described to have ASPS in femoral region. A combined chemotherapy (radiotherapy, hyperthermia and chemotherapy) was performed before operation. Tumor wide resection was carried out, however, a metastatic lesion revealed in the lung continued to increase in number and size during the progress after operation, however, it decreased in size and number after several years [12].

In 1988, Pang and colleagues have report a case of female patient with ASPS who survived for over 9 years after detection of pulmonary metastases regressed when she was treated with oral and topical Chinese herbs. She had excision of a right buttock mass in 1975. She was given cytotoxic chemotherapy, but there was no radiological response. Thereafter; she took herbal mixture from March 1979 to July 1982. Chest X-rays in 1983 showed complete disappearance of some shadows and shrinkage of others. [13]

In a study conducted by Kebudi R. et al between 1989 and 2002, the incidence of brain metastases in extracranial solid tumors was studied in pediatric age group. Only 16 out of 1100 children with extracranial solid tumors developed brain mets. One of the 16 was found to be alveolar soft part sarcoma. [14]

The aim of publishing our case, the second of its type in the literature, was to encourage the study of biological behavior of ASPS as well as common molecular characteristics that could be present in other tumors that tend to regress alone.

## Conclusion

ASPS is a rare type of sarcomas that affect primarily the lower limbs. This tumor does rarely metastasize to the gastrointestinal tract. We can only speculate on the unusual spontaneous regression reported in this case. We do not have any solid information of a cytotoxic effect of the herb used, so we speculate that the regression was spontaneous probably mediated by the immunologic system of the host. However, this effect was short termed as the patient has died from further progression and metastasis of the tumor.

## Consent

Written informed consent was obtained from the next of kin for reporting of this case, the copy of consent is available with editor in chief.

## Competing interests

The authors declare that they have no competing interests.

## Authors' contributions

MB and ARA were involved in the concept and design, preparation of draft and editing of final manuscript. Both authors read and approved the final manuscript.
